# Single center experience with ALPPS and timing with stage 2 in patients with fibrotic/cirrhotic liver

**DOI:** 10.1007/s13304-024-01782-x

**Published:** 2024-03-17

**Authors:** Kuo-Chen Hung, Hao-Ping Wang, Wei-Feng Li, Yu-Cheng Lin, Chih-Chi Wang

**Affiliations:** 1 Department of Surgery, Kaohsiung Chang Gung Memorial Hospital, 123 Ta-Pei Road, Niao-Song, Kaohsiung, 833 Taiwan; 2 Chang Gung University College of Medicine, Taoyüan, Taiwan

**Keywords:** ALPPS, Liver fibrosis/cirrhosis, Hepatocellular carcinoma, Viral hepatitis, Remnant liver volume, Liver regeneration

## Abstract

Associating liver partition and portal vein ligation for staged hepatectomy (ALPPS) is a novel procedure for major resection in patients with insufficient future liver remnant (FLR). Effective FLR augmentation is pivotal in the completion of ALPPS. Liver fibrosis/cirrhosis associated with chronic viral hepatitis impairs liver regeneration. To investigate the augmentation of FLR in associating ALPPS between patients with fibrotic/cirrhotic livers (FL) and non-fibrotic livers (NFL) and compare their short-term clinical outcomes and long-term survival. Patients were divided into two groups based on the Ishak modified staging: non-fibrotic liver group (NFL, stage 0) and fibrotic/cirrhotic liver group (FL, stage 1–5/6). Weekly liver regeneration in FLR, perioperative data, and survival outcomes were investigated. Twenty-seven patients with liver tumors underwent ALPPS (NFL, *n* = 7; FL, *n* = 20). NFL and FL patients had viral hepatitis (28.6% [*n* = 2] and 95% [*n* = 19]), absolute FLR volume increments of 134.90 ml and 161.85 ml (*p* = 0.825), and rates of hypertrophy were 16.46 ml/day and 13.66 ml/day (*p* = 0.507), respectively. In the FL group, baseline FLR volume was 360.13 ml, postoperatively it increased to a plateau (542.30 ml) in week 2 and declined (378.45 ml) in week 3. One patient (3.7%) with cirrhotic liver (stage 6) failed to proceed to ALPPS-II. The overall ALPPS-related major complication rate was 7.4%. ALPPS is feasible for fibrotic liver patients classified by Ishak modified stages ≤ 5. After ALPPS-I, 14 days for FLR augmentation seems an appropriate waiting time to reach a maximum FLR volume in these patients.

## Introduction

Liver resection is the potential curative treatment for hepatocellular carcinoma (HCC) and colorectal metastases (CRLM) [1, 2]. Insufficient future liver remnant (FLR) remains a big hurdle for patients who require major hepatectomy and predisposes them to develop post-hepatectomy liver failure and potential mortality [3]. To overcome insufficient FLR post-hepatectomy, augmentation of FLR by portal vein embolization (PVE) [4] was the conventional approach to improve the safety of major hepatectomy. Recently, associating liver partition and portal vein ligation for staged hepatectomy (ALPPS) has been a popular novel approach for FLR augmentation, which induces a rapid increase of FLR volume (40–160% within 1–2 weeks) [5–8].

Currently, CRLM is the main indication for ALPPS. There are few and conflicting data of ALPPS for fibrotic/cirrhotic liver patients with chronic viral hepatitis [9, 10]. The aim of this study was to investigate the augmentation of FLR in ALPPS between patients with fibrotic/cirrhotic livers and non-fibrotic livers, and to compare their short-term clinical outcomes and long-term survival.

## Materials and methods

This is a retrospective study of patients receiving ALPPS at Kaohsiung Chang Gung Memorial Hospital and Chiayi Chang Gung Memorial Hospital between January 2013 and December 2017. Patients with acceptable cardiopulmonary function and acceptable anesthesiological risks were considered for liver resection.

Patients were divided into two groups according to their pathological findings based on Ishak modified staging: [11] non-fibrotic liver group (NFL, stage 0) and fibrotic/cirrhotic liver group (FL, stage 1–5/6). Patients with liver tumors underwent relevant investigations and radiologic evaluation, including a fibroscan, contrast-enhanced computed tomographic angiography, and measurement of liver volumes.

### Patients

According to the preoperative image-based volumetric planning, an FLR volume** ≤ **35% of standard liver volume (SLV) in a non-cirrhotic liver patient or ≤ 40–45% of SLV in patients with Child–Pugh A liver cirrhosis or steatosis was considered a potential indication to perform the ALPPS procedure. Indocyanine green retention rate (ICG) should be ≤ 25% at 15 min. The indications of major resection for liver tumors, which result in insufficient FLR included a huge liver mass with size ≥ 8 cm, multiple tumors, and a central location with inevitable major vascular resection. Patients with untreatable liver tumors in the FLR, extrahepatic metastases, and medical contraindications to major hepatectomy were excluded from ALPPS surgery.

The severity of perioperative morbidity and mortality was graded according to the Clavien-Dindo classification [12]. Major complications were defined as Clavien grade ≥ 3B. Grading of post-hepatectomy liver failure (PHLF) was defined according to the International Study Group of Liver Surgery (ISGLS) classification [3].

### Liver volumetry

FLR and total liver volume were gauged before (baseline FLR0) and weekly after ALPPS-I from computed tomography (CT) scans. SLV was calculated by the Urata formula [13]. FLR volumetric changes before and after ALPPS-I were expressed in the form of absolute gain in volume increment and changes in the FLR/SLV ratio. Absolute kinetic growth rate (AKGR) was assessed by the change in volume divided by the number of waiting days to CT volumetry (ml/day). The relative kinetic growth rate was expressed as ratio of AKGR/FLR0.

### Surgical techniques

Liver resections and anatomy were defined according to the Brisbane 2000 Terminology and COUINAUD segmentation of the liver [14, 15].

### ALPPS-I

The meticulous techniques of hepatectomy have been described in a previous paper [16]. The hanging maneuver was carried out through dissecting the retrohepatic area at the caudal end, coursing in a cephalad fashion toward the space between the right and middle hepatic veins. An angled long aortic clamp was gently inserted and advanced in a cephalad direction at the 10–11 o’clock position toward the dissected space between the right and middle hepatic veins. When the tip of the clamp reached the space between the veins, a Penrose tube tape was clenched in the tip of the clamp [17]. The hilar structures were explored, and a vascular loop was passed around the right hepatic artery (HA). The right portal vein (PV) was identified and clamped followed by ligation with silk after the liver partition. A nearly complete or complete parenchymal split [18] with a Cavitron Ultrasonic Surgical Aspirator (CUSA) to the level of the hilar plate just above the inferior vena cava was decided not to induce uncontrollable bleeding to avoid a premature resection of the tumor-bearing liver and possible postoperative bleeding complicated with infection, which would result in a premature second laparotomy. A perioperative Doppler ultrasound check was performed to ensure patency of the intrahepatic vessels except the right PV. A sterile plastic bag (IV bag) was applied in the subhepatic space between the liver and gastroduodenal tracts and transection space to decrease adhesion. At the end of the procedure, the right HA, RPV, main PV, and right intrahepatic duct were encircled with vascular loops to facilitate their identification during ALPPS-II. A cholecystectomy was performed, and a transcystic tube was inserted to check biliary anatomy, followed by marking the transection line of the biliary tract for cutting the bile duct in ALPPS-II and leakage at the end of the liver partition. Two drain tubes were placed in the subphrenic and raw surfaces of the liver. We avoided excessive dissection of the surrounding tissues to prevent adhesions.

### ALPPS-II

During the second stage, the abdomen was entered through the previous incision. Intra-abdominal fluid was collected for routine microbiological examinations. The tumor-bearing liver was resected after transecting the right hepatic duct, right HA, right PV, and right hepatic vein. Possible clots from the stump of the right PV were flushed followed by appropriate suturing. A large amount of saline water was irrigated through the entire abdominal cavity with drains at the right subphrenic and raw surfaces of the transection.

### Histopathology

Hematoxylin–eosin-stained slides and immunohistochemical analysis of specimens were reviewed and fibrosis was scored according to the Ishak modified staging. A score of 0–6 was given according to the degree of fibrosis: 0 (no fibrosis), 1–5 (mild–severe fibrosis), and 6 (cirrhosis) [11].

### Follow-up

Once discharged home, the patients were followed at our outpatients’ clinic once every month for the first 3 months and thereafter, once every 3 months.

### Statistical analysis

All continuous variables were expressed as medians with interquartile ranges and compared using the Mann–Whitney *U* test. Categorical variables were compared with Fisher's exact test. The Kaplan–Meier curve was calculated for survival analysis and comparison between groups was tested by the Log-rank test. Spearman’s correlation was used to measure association between the fibroscans and liver volumes. The change in liver volume, including pre-ALPPS-I and -II FLR, pre-ALPPS-I and -II FLR/SLV, increment in FLR/SLV, absolute increment in FLR volume, absolute increment in FLR volume/FLV0, increment in FLR volume /FLV0 per day, rate of hypertrophy, and increment in FLR/SLV per day during the follow-up time were calculated using generalized estimating equations (GEE). GEE were also used to compare these changes between the fibrosis/cirrhosis and normal liver groups. The 2-sided *p* values < 0.05 were considered statistically significant. Statistical analysis was performed using SPSS Statistics for Windows 25.0 (IBM Corp., Armonk, NY, USA).

The institutional review board gave approval for this study (No. 201902147B0). Informed consent was obtained from all patients before the procedures. All cases were reviewed and discussed by the HCC multidisciplinary committee.

## Results

### Demographics

Twenty-seven patients with liver tumors underwent ALPPS. Patients’ demographics are presented in Table 1. The NFL group had 7 patients and the FL group had 20 patients. In the NFL group, 28.6% (*n* = 2) of patients and in the FL group 95% (*n* = 19) of patients had viral hepatitis. One patient (3.7%) who had a cirrhotic liver with Ishak modified stage 6, failed to proceed to ALPPS-II due to inadequate FLR hypertrophy. Liver fibrotic stage showed significance with fibroscan (*p* = 0.012) between the groups. Of the 27 patients who participated in this study, 96.2% (*n* = 26) completed ALPPS-II surgery.

**Table 1:** Clinicopathological data

	Non-fibrotic/cirrhotic liver *n* = 7	Fibrotic/cirrhotic liver *n* = 20	*p*
M:F	4:3	19:1	0.042
Age	58 (49–65)	64 (63–71)	0.107
BMI	21.82 (21.22–25.96)	23.82 (22.60–26.15)	0.245
Hepatitis			
HBsAg positivity (HBV)	1	9	0.002
Anti-HCV positivity (HCV)	1	9	
HBV&HCV positive	0	1	
HBV&HCV negative	5	1	
Liver tumor	7	20	0.026
HCC	4	16	
CRLM	3	0	
CCC	0	3	
FNH	0	1	
ICG clearance rate (15 min)	8.60 (4.70–9.20)	10.30 (5.33–18.08)	0.293
Ishak modified stage of fibrosis (stage) 0/1/2/3/4/5/6	7/0/0/0/0/0/0	0/1/2/7/5/4/1	
Child–Pugh Score	3 (3–3)	3 (3–3)	0.554
Fibroscan	5.70 (4.45–7.20)	9.05 (6.68–10.68)	0.012
Waiting time from ALPPS-I to II (day)	12 (10–14)	13 (12–14)	0.229
Blood loss, mL			
ALPPS-I	120.0 (60.0–150.0)	150.0 (100.0–287.5)	0.175
ALPPS-II	150.0 (100.0–350.0)	100.0 (50.0–250.0)	0.619
Total blood loss	350.0 (160.0–470.0)	330.0 (200.0–650.0)	0.621
Overall complication rate	15% (2/7)	36.8% (7/20)	1.000
90-days mortality rate	0	0	–
Hospital stay, day	31.0 (21.0–42.0)	24.0 (22.0–35.0)	0.707

*BMI* body mass index, *HCC* hepatocellular carcinoma, *CRLM* colo-rectal liver metastasis, *CCC* cholangiocarcinoma, *FNH* focal nodular hypertplasia

### Liver volumetry

**Fig. 1:**
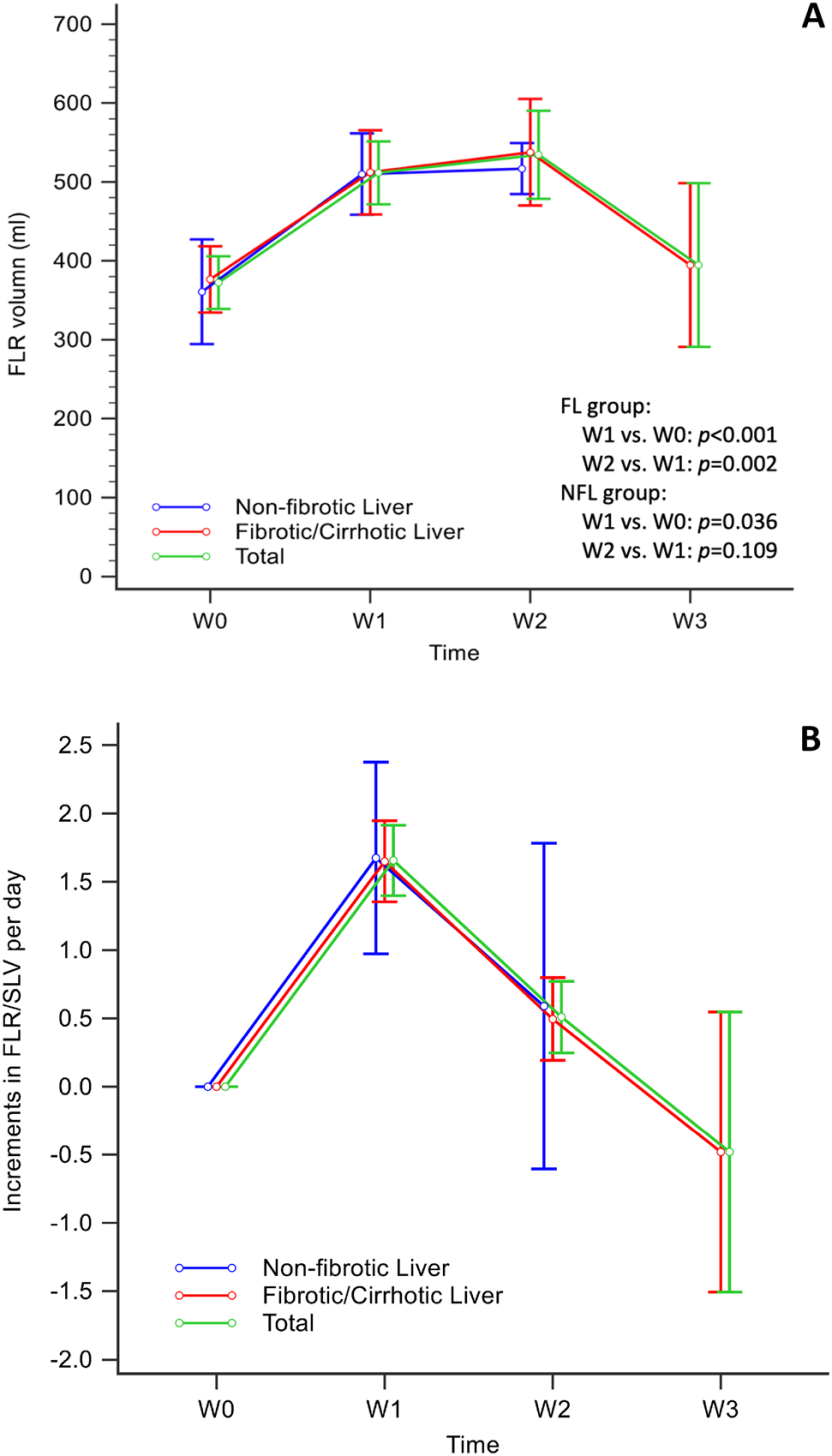
**A** Future liver remnant volume increased rapidly in the first week after associating liver partition and portal vein ligation for staged hepatectomy (ALPPS-I) and slowed to a plateau in postoperative week 2 in the non-fibrotic liver group. In fibrotic liver group, liver regeneration continued and peaked in postoperative week 2 and the hypertrophic liver began to shrink during postoperative week 3. **B** Increments in FLR/SLV per day reached the highest level in postoperative week 1 then declined. Hypertrophic livers began to shrink during postoperative week 3. Increments in FLR/SLV per day in weeks 1 and 2 were 1.43% and 0.43% (*p* = 0.105), respectively in the non-fibrotic liver group and 1.47% and 0.42% (*p* < 0.001), respectively in the fibrotic liver group. *FLR* future liver remnant, *SLV* standard liver volume, *W0* pre-ALPPS surgery, *W1* week 1 after associating liver partition and portal vein ligation for staged hepatectomy (ALPPS-I) operation, *W2* week 2 after ALPPS-I operation, *W3* week 3 after ALPPS-I operation

The data on preoperative FLR volume (FLR0) and postoperative FLR volume changes are listed in Table 2. FLR0 volumes were 357.80 ml and 360.13 ml (*p* = 0.543) and pre-ALPPS-II volumes were 512.20 ml and 533.70 ml (*p* = 0.803) in the NFL and FL groups, respectively. The FLR volume increased after ALPPS-I, reached a peak in week 2, and then declined. In the FL group, liver regeneration continued and liver volume increased significantly until week 2 (*p* = 0.002) (Fig. 1A). Absolute increments in FLR volume were 134.90 ml and 161.85 ml (*p* = 0.825) and rates of hypertrophy were 16.46 ml/d and 13.66 ml/d (*p* = 0.507), respectively in the NFL and FL groups. Increments in FLR/SLV per day in week 1 and week 2 were 1.43% and 0.43% (*p* = 0.105) in the NFL group and 1.47% and 0.42% (*p* < 0.001) in the FL group (Fig. 1B). FLR volume changes and kinetic growth rates between the groups showed no significant difference. FLR volume changes and increments in FLR/SLV per day between weeks 1 and 2 differed significantly in the FL group (Fig. 1A, B).

**Table 2:** Sequential volumetry changes of pre-ALPPS and post-ALPPS in patients with non-fibrotic or fibrotic/cirrhotic livers

	Non-fibrotic liver (*n* = 7)	Fibrosis/cirrhosis liver (*n* = 20)	*p*
SLV, ml	1154.06 (1085.41–1331.50)	1217.38 (1156.81 to 1282.54)	0.472
Pre-ALPPS I FLR (FLV0), mL	357.80 (290.90–388.00)	360.13 (321.66 to 413.58)	0.543
Post-ALPPS I FLR, 1st week, mL	496.70 (458.70–552.20)	495.16 (434.54 to 572.13)	0.825
Post-ALPPS I FLR, 2nd week, mL	512.20 (506.90–531.70), *n* = 3	542.30 (424.10 to 605.88), *n* = 16	0.823
Post-ALPPS I FLR, 3rd week, mL	–	378.45 (343.15 to 462.63), *n* = 4	–
pre-ALPPS II FLR, mL	512.20 (506.90–552.20)	533.70 (426.93 to 605.88)	0.803
Pre-ALPPS I FLR/SLV, %	30.27 (22.37–37.29)	30.01 (27.19 to 34.67)	0.956
Pre-ALPPS II FLR/SLV, %	46.70 (39.55–47.93)	44.40 (36.07–48.96)	0.472
Increment in FLR/SLV, %	12.43 (10.0–17.91)	13.18 (9.35 to 15.79)	0.740
Absolute increment in FLR volume, mL	134.90 (115.20–237.60)	161.85 (112.05 to 200.74)	0.825
Absolute increment in FLR volume/FLV0, %	36.26 (28.20–82.78)	43.20 (29.65 to 55.58)	0.868
Increment in FLR volume /FLV0 per day, % (RKGR)	3.34 (3.13–6.48)	3.52 (2.66 to 5.02)	0.619
Rate of hypertrophy, mL/d (AKGR)	16.46 (11.24–19.80)	13.66 (9.06 to 19.81)	0.507
Increment in FLR/SLV per day, %, (median (min–max)	1.38 (0.84–1.96)	1.11 (− 0.07 to 2.29)	0.268
Increment in FLR/SLV per day, 1st week, %, (median (min–max)	1.43 (0.84–3.01)	1.47 (0.72 to 2.87)	1.000
Increment in FLR/SLV per day, 2nd week, %, (median (min–max)	0.43 (0.21–1.13), *n* = 3	0.42 (− 0.14 to 2.00), *n* = 16	0.695
Increment in FLR/SLV per day, 3rd week, %, (median (min–max))	–	− 0.38 (− 1.27 to 0.10), *n* = 4	–

Values are presented as medians with inter-quartile ranges (IQR) or range (minimum–maximum). ALPPS indicates associating liver partition and portal vein ligation for staged hepatectomy

*FLR* future liver remnant, *FLV0* baseline future liver remnant volume, *AKGR* absolute kinetic growth rate, *RKGR* relative kinetic growth rate

### Perioperative outcomes and survival

The perioperative data is summarized in Table 1. The median waiting time for FLR augmentation from ALPPS-I to ALPPS-II was 12 days and 13 days in NFL and FL groups, respectively (*p* = 0.229). The waiting time was ≤ 9 days for 15.3% patients (*n* = 4), 10–16 days for 73.1% patients (*n* = 19), 17–22 days for 11.5% patients (*n* = 3). PHLF grade A/B on postoperative day (POD) 5 after ALPPS-II, according to the ISGLS classification were 14.29%/0 and 21.05%/15.79% in the NFL and FL groups, respectively. There was no PHLF grade C.

**Fig. 2:**
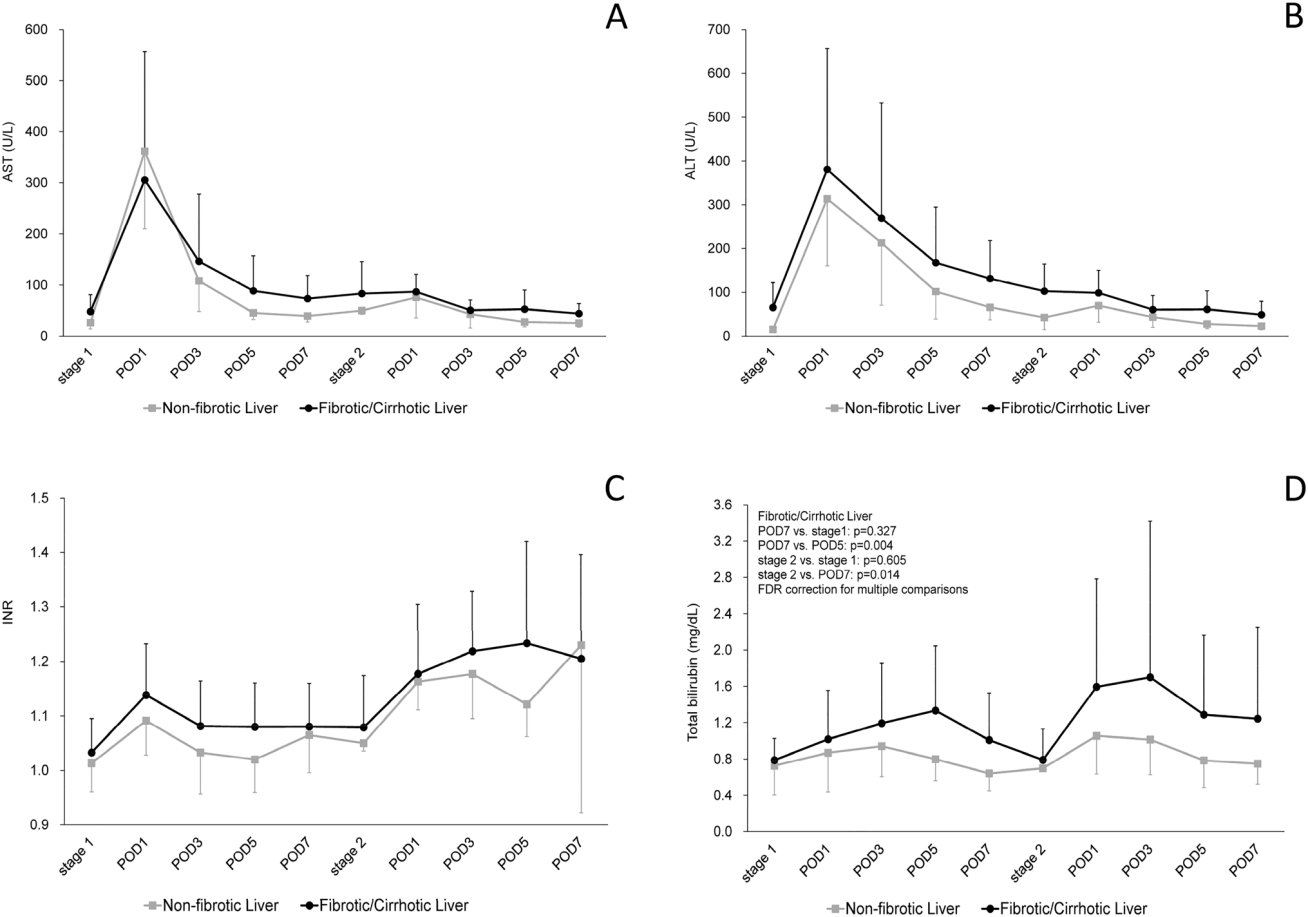
Postoperative course of biochemical blood tests following ALPPS-I and ALPPS-II: **A** AST. **B** ALT, aminotransferase levels (AST, ALT) following ALPPS-I peaked on POD 1, and then decreased. After ALPPS-II, aminotransferase levels did not show fluctuation. **C** INR following ALPPS-I peaked on POD 1 and returned to normal values on POD 3 in both groups (**C**). After ALPPS-II, INR peaked on POD 3 in the NFL group and on POD 5 in the FL group. **D** Total bilirubin peaked on POD 3 in the NFL group and on POD 5 in the FL group, returning to baseline levels (pre-ALPPS-I) on POD 5 in the NFL group and on the day of ALPPS-II in the FL group (*p* = 0.065) after ALPPS-I. Transient impaired liver functions during the first week following ALPPS-II may be attributed to the loss of deportalized livers. *AST* aspartate aminotransferase, *ALT* alanine aminotransferase, *ALPPS* associating liver partition and portal vein ligation for staged hepatectomy, *POD* postoperative day, *INR* international normalized ratio; stage 1, the day before ALPPS-I; stage 2, the day of ALPPS-II; *FL* fibrotic liver, *NFL* non-fibrotic liver

The postoperative course of biochemical blood tests following ALPPS-I and ALPPS-II are presented in (Fig. 2A–D). After ALPPS-I, median levels of bilirubin peaked on POD 3 in NFL patients and on POD 5 in FL patients and returned to pre-ALPPS-I baseline levels on POD 5 in NFL patients and on the day of ALPPS-II (approximately POD 14) in FL patients (*p* = 0.065) (Fig. 2D). After ALPPS-II, serum bilirubin increased and peaked on POD 1 in NFL patients, and POD 3 in FL patients (Fig. 2D). INR levels following ALPPS-I peaked on POD 1 and returned to normal values on POD 3 in both groups (Fig. 2C). After ALPPS-II, INR peaked on POD 3 in the NFL group and on POD 5 in the FL group (Fig. 2C). Transient impaired liver functions during the first week following ALPPS-II may be attributed to the loss of deportalized livers. Following ALPPS-I, aminotransferase levels (AST, ALT) peaked on POD 1 and then decreased. After the ALPPS-II operation, aminotransferase levels did not fluctuate (Fig. 2A, B).

**Fig. 3:**
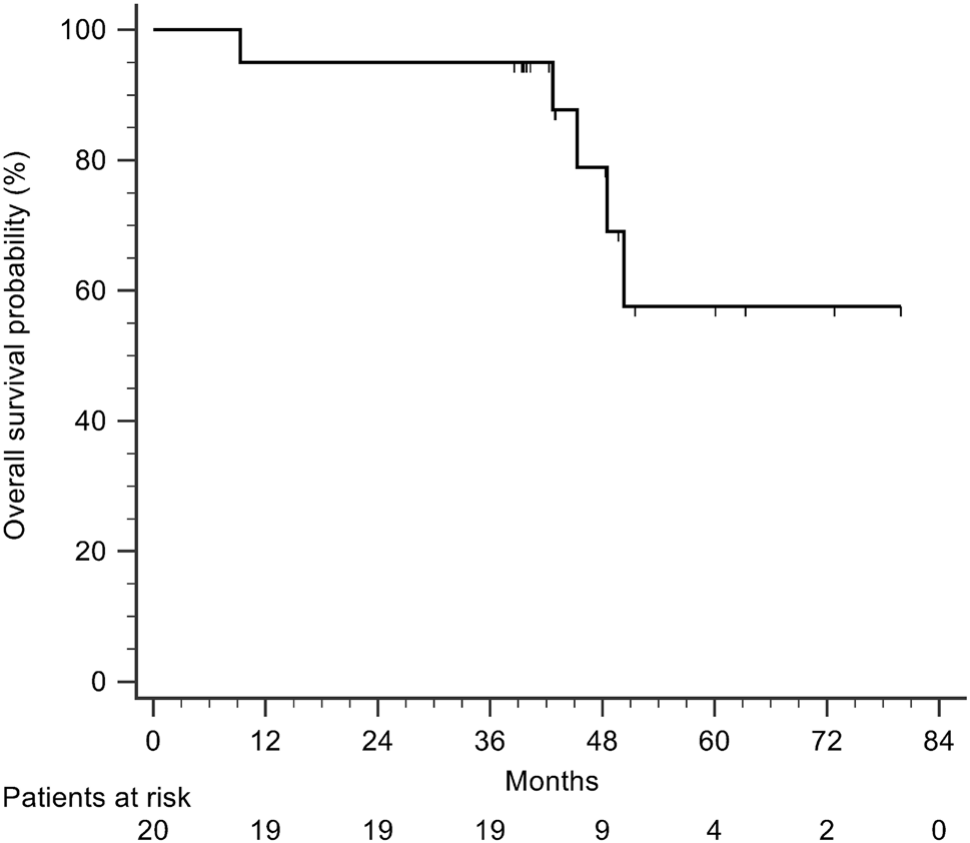
Overall survival of patients with hepatocellular carcinoma after associating liver partition and portal vein ligation for staged hepatectomy (ALPPS)

ALPPS-related complication rates after ALPPS-I and ALPPS-II were 0% and 28.6%, and 15% and 36.8% in the NFL and FL groups, respectively. The overall major complication (≥ IIIb) rate was 7.4% (2/27). No difference in total perioperative blood loss, complication rates, and hospital days was observed between the two groups. There were no patient deaths by 90 days after the surgery. Median follow-up time was 41.7 (38.6–50.3) months. The OS rates of HCC patients at 1, 3, and 5 years were 95.0%, 95.0%, and 57.5%, respectively (Fig. 3).

## Discussion

The liver regeneration process is divided into three phases [19]. The early phase is rapid volume increase, which occurs during the first 2 postoperative weeks. The second phase is volume decline at 1–2 months after hepatectomy. The third phase is a slow increase in volume, until the liver volume/body weight ratio plateaus. In living donor liver transplantations, the residual liver volume after a left lateral or left hepatectomy (20–35% liver loss) increased gradually for a 1 week and then decreased from 1 to 3 months, reaching ~ 90% of the preoperative liver volume at 1 year [20]. For ALPPS, Truant’s study with 80.6% of patients with CRLM, the hypertrophy rate of the FLR was slower and tended to a plateau beyond 7 days [21]. ALPPS-II followed usually within a median of 7 days after ALPPS-I, in the context of CRLM [6]. Nonetheless, the liver volume increment and growth rate after hepatectomy showed an inverse correlation with the severity of chronic liver diseases [22]. The clinical implication is that a longer period to ALPPS-II would be necessary for fibrotic/cirrhotic livers to allow adequate FLR volume and function recovery. There is no evidence to demonstrate the duration of the wait for a safe FLR in patients with FL. In the FL group of our study, the liver regeneration rate (increments in FLR/SLV per day) was the fastest in week 1, slowed in week 2, and showed negative growth in week 3 (Fig. 1B). The volume of FLR reached its highest level in week 2, after which the hypertrophic liver shrank (Fig. 1A). Serum bilirubin and INR are well-known measures of hepatic function and represent components of ISGLS grading of PHLF [3]. The FL group took 2 days longer than the NFL group in the liver function recovery of serum bilirubin after ALPPS-I. From the liver growth curve in our study, a waiting period of 14 days seems to reach a maximum FLR volume with restored liver function in fibrotic livers.

In our study, the median day of interval between the two stages was 13 days for the FL group. This is longer than previous studies. We assumed that volumetric liver regeneration was not equal to functional liver regeneration. Therefore, we waited for 2 weeks with most patients. This idea was compatible with Olthof et al. [23]. Their study aimed to evaluate functional liver regeneration in contrast to volumetric liver regeneration in ALPPS, using technetium-99 m hepatobiliary scintigraphy and CT volumetry, respectively. The results revealed volumetry overestimates liver function as measured by hepatobiliary scintigraphy and may be responsible for the high rate of liver failure.

The key feature of ALPPS is the rapid regeneration of FLR after ALPPS-I, when compared with conventional two-stage hepatectomy with portal vein embolization (PVE). The presumed molecular pathways behind this unprecedented liver regeneration remain unclear. Accelerated liver growth may be associated with upregulated circulating inflammatory and growth factors under the effect of a parenchymal split (PS) [24].

We performed a complete PS in our study for two reasons. Firstly, in NFL patients, a partial PS of ≤ 50% seems to be equally effective with complete PS [25]; however, a complete PS induced more rapid FLR hypertrophy than a partial PS without increased perioperative risk in chronic liver diseases [18]. The second reason was the surgical technique consideration. The ALPPS procedure is a major operation that requires a high level of expertise. Early experience of ALPPS has been hampered by the high incidence of postoperative complications and the high incidence of mortality [26]. Complete PS provided ALPPS-II a simpler procedure [18], only a division of the diseased liver inflow pedicle and hepatic vein. We placed a saline bag on the dissection surface, which prevented cut parenchymal adhesions and rendered a cleaner operative view. In addition to the meticulous surgical technique, a complete PS and saline bag placement contribute to the uncommon morbidity of bile leakage and infection (7.4%, *n* = 2) in ALPPS-I of our series. Elevated aminotransferases (AST, ALT) can be indicators of liver damage. Aminotransferase levels did not fluctuate during ALPPS-II in our study (Fig. 1A, B), implying the second operation did not damage the residual liver parenchyma, an attribution to complete PS in ALPPS-I.

**Fig. 4:**
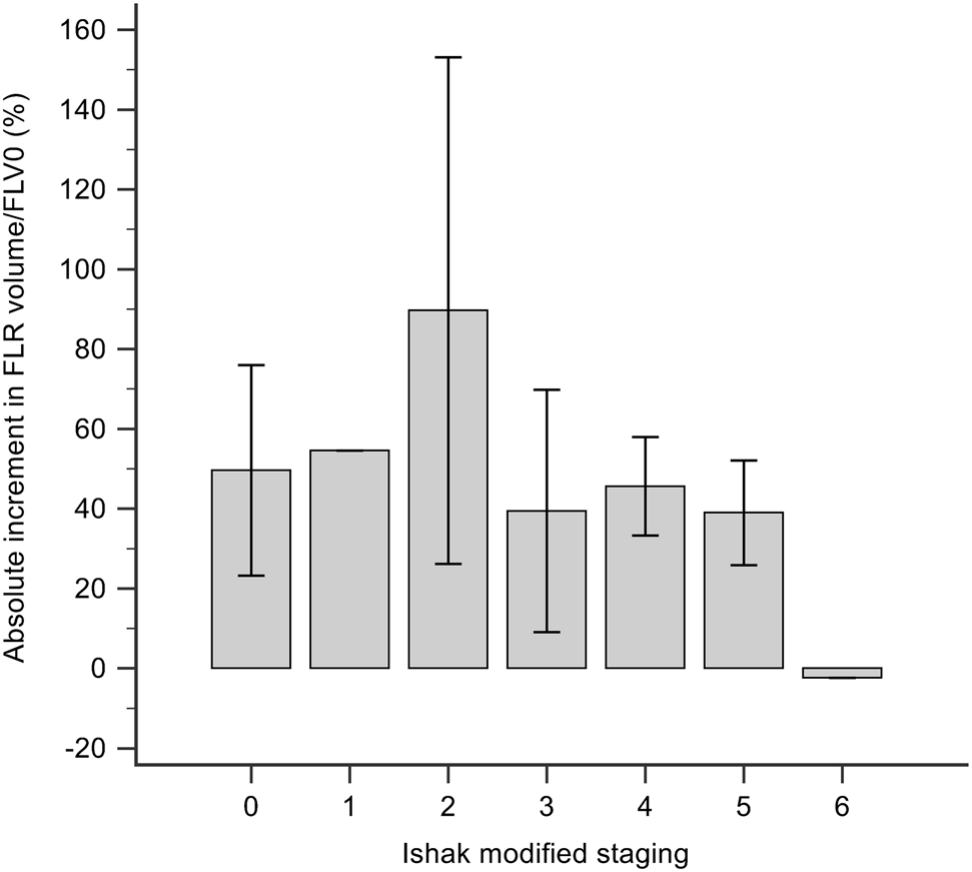
Absolute increment in FLR volume/FLV0 (%) after ALPPS. The FLR hypertrophy rate did not show a significant correlation with the severity of liver fibrosis and cirrhosis (*p* = 0.868). There seems a trend that fibrosis more than moderate stage (2/3) had a negative impact on the hypertrophy of the FLR. *ALPPS* associating liver partition and portal vein ligation for staged hepatectomy, *FLR* future liver remnant, *FLV0* baseline future liver remnant volume

The definition of liver fibrosis/cirrhosis is variable. A liver biopsy is an important method of assessing the severity of liver disease, in terms of grade and stage. Several methods [11, 27–29] are currently used to express the grade and stage of chronic hepatitis. Ishak modified staging [12] with 7 stages (0–6) provides more information than other fibrosis scores with only 5 stages (0–4) [27–29]. Therefore, it was used to distinguish between early cirrhosis/incomplete cirrhosis (stage 5) and complete cirrhosis (stage 6). In their multicenter study of 35 patients with HCC, D’Haese et al. [9] showed that hypertrophy and kinetic growth varied significantly with the severity of fibrosis. A similar pattern was seen in our study, though it was not statistically significant (*p* = 0.868), we could still see a trend that more than moderate stage (2/3) fibrosis was a negative impact on the hypertrophy of the FLR (Fig. 4). In our series, one patient with Ishak modified stage 6 could not proceed to the ALPPS-II operation due to inadequate FLR augmentation. The reason being liver regeneration would be hindered in the completely cirrhotic liver.

Schlitt (2007), performed the first ALPPS procedure [30]. During the past years, improved patient selection and refinements in operative techniques have reduced morbidity and mortality rates. ALPPS which remains a very difficult procedure and should only be performed in reference centers. The limitation of this study is a retrospective and observational research with a small sample size. It needs more prospective randomized controlled trials to clarify.

Liver regeneration is impaired in patients with fibrotic livers and a longer period is required for restoration of liver function after the hepatectomy. The findings of the present study revealed that 14 days are an appropriate waiting time for FLR augmentation after ALPPS-I, to reach a maximum volume of FLR with restored liver function in patients with fibrotic livers (Ishak modified stage ≤ 5).

## Data Availability

The classification of ISHAK is described on reference [11]. The ISHAK data of our patients are showed on Table 1-Clinicopathological data.
